# Hierarchical Association Coefficient Algorithm: New Method for Genome-Wide Association Study

**DOI:** 10.1177/1176934317713004

**Published:** 2017-08-31

**Authors:** Bongsong Kim

**Affiliations:** Department of Agronomy, Iowa State University, Ames, IA, USA

**Keywords:** Hierarchical Association Coefficient, HA-coefficient, Genome-wide association study, GWAS, QTL

## Abstract

Hierarchical association coefficient algorithm calculates the degree of association between observations and categories into a value named *hierarchical association coefficient* (HA-coefficient) between 0 for the lower limit and 1 for the upper limit. The HA-coefficient algorithm can be operated with stratified ascending categories based on the average of observations in each category. The upper limit refers to a condition where observations are increasingly ordered into the stratified ascending categories, whereas the lower limit refers to a condition where observations are decreasingly ordered into the stratified ascending categories. An HA-coefficient represents how close an observed categorization is to the upper limit, or how distant an observed categorization is from the lower limit. To demonstrate robustness and reliability, the HA-coefficient algorithm was applied to 3 different simulated data sets with the same pattern in terms of the association between observations and categories. From all simulated data sets, the same result was obtained, indicating that the HA-coefficient algorithm is robust and reliable.

## Introduction

A genome-wide association study (GWAS) is an analysis of categorical data. The GWAS data consist of categorical variables (categories) with patterned DNA sequences and a quantitative variable (observations) with real numbers for a trait of interest. This article introduces a new method for measuring association between categories and observations, named the *hierarchical association coefficient* (HA-coefficient) algorithm. The algorithm measures the association between categories and observations based on the degree of variance among the averages for all categories. If averages across *n* categories are similar, this suggests a situation where observations are randomly distributed into categories. If averages across different categories are clearly different, this suggests a situation where observations are assigned into categories by some criterion, and it can be said that categories and observations are associated. This foundation also applies to the *F* test which calculates a *P* value referring to the degree of variance among averages for all categories and is widely used for GWAS.^1–4^

To measure the association between categories and observations, the HA-coefficient algorithm uses 2 sorting extremes: (1) observations being increasingly sorted into stratified ascending categories (HA-coefficient = 1), and (2) observations being decreasingly sorted into stratified ascending categories (HA-coefficient = 0). Note that the stratified ascending categories means a condition where observed categories are aligned in ascending order based on the average of observations in each category. The sorting extremes are conditions where the degree of variance among the averages for all categories are maximized. Meanwhile, the *F* test calculates a ratio of intercategorical variability to intracategorical variability, in which the greater the ratio, the more variance among the averages for all categories is found.^5^ Simulations revealed that the HA-coefficient algorithm and *F* test produce similar results. The *F* test is a method for a hypothetical test, whereas the HA-coefficient algorithm calculates an objective measurement.

## Theory and Methods

### Hierarchical association distance

Given the whole population set has 2 or more members and is categorical, let us make the following conventions:

1. Every member has a positive real number as an observation.
2. Every member has a categorical identifier.
3. Averages of observations in different categories are different.

Then, the categories can be stratified based on the average of observations. On this basis, let us define:

#### Definition 1

“Hierarchical” means that all categories are stratified in ascending order based on the average of each category.

#### Definition 2

Suppose that all categorical boundaries in hierarchical stratification are fixed, and observations are permutable. “Top categorization” means a condition in which observations are arranged in ascending order in each category leading to ascending order across all categories.

#### Definition 3

Suppose that all categorical boundaries in hierarchical stratification are fixed, and observations are permutable. “Bottom categorization” means a condition in which observations are arranged in descending order in each category leading to descending order across all categories.

#### Definition 4

“Hierarchical association coefficient” means a proportion representing how close the top and observed categorizations are, or how distant the bottom and observed categorizations are.

#### Definition 5

Suppose that *n* categories are stratified in ascending order based on the average of each category from left to right, in which *n* = the number of all categories. There are *n* − 1 categorical boundaries. At each categorical boundary, we can make 2 categories by collapsing the other categorical boundaries. Let us call the result “hierarchical binary categorization” and designate the sum of the right subset as *x*1 and the sum of the left subset as *x*2 at any categorical boundary. The *x*1 is a representative value for a respective hierarchical binary categorization.

**Figure 1.**
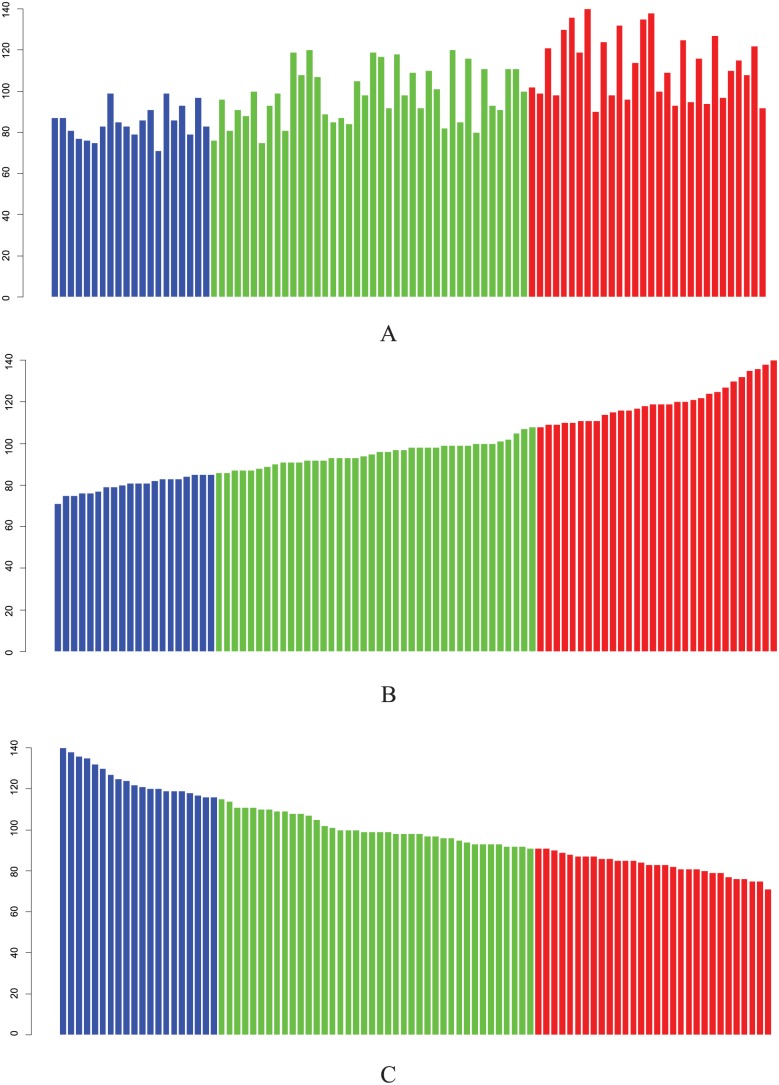
Three categorizations (A, B, C) including 3 categories (blue, green, red). Each bar represents an observation. All categorizations contain the same observations. (A) Observed categorization in which categories are sorted in ascending order based on the average of each category. (B) The top categorization. (C) The bottom categorization.

Regarding Definitions 1 to 3, graphical instructions are shown in Figure 1. Definition 5 always assures that (1) *x*1 in the top categorization is equal to or greater than *x*1 in the observed categorization, and (2) *x*1 in the bottom categorization is equal to or less than *x*1 in the observed categorization. The use of *x*1 allows us to quantify the hierarchical association distance by substituting *x*1 as a value for an observed categorization for *x* in the following equation:

(1) $$d_{x}=\frac{g1}{g2}\left(\frac{y}{x}-1\right)$$

where *x* is the variable, $d_{x}$ is the hierarchical association distance given *x, g*1 is the *x*1 in the top categorization, *g*2 is the *x*2 in the top categorization, and *y* is the sum of all observations.

Equation 1 can be derived as follows:

y=g1+g2=r1+r2

Substitute *x* as a variable for *r*1 so that *r*2 = *y* − *x*. Then,

$$d_{x}=\frac{g1}{r1}\cdot \frac{r2}{g2}=\frac{g1}{g2}\cdot \frac{y-x}{x}=\frac{g1}{g2}\left(\frac{y}{x}-1\right)$$

where *y* is the sum of all observations, *g*1 is the *x*1 in the top categorization, *g*2 is the *x*2 in the top categorization, *r*1 is the *x*1 in the observed categorization, *r*2 is the *x*2 in the observed categorization, *x* is the variable, and *d_x_* is the hierarchical association distance given *x*.

**Figure 2.**
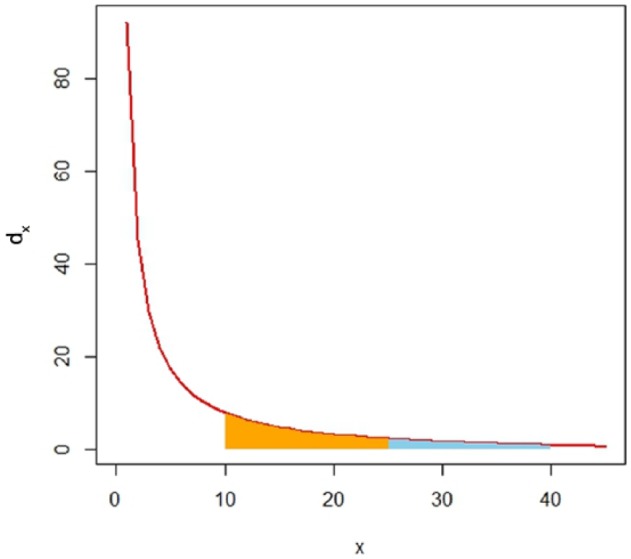
A curve for $d_{x}=(40/30)((70/x)-1)$. The whole colored area is delimited by two *x*1s in the bottom and top categorizations. The orange area is delimited by two *x*1s in the bottom and observed categorizations.

It is always true that 1≤(g1/r1) and 1≤(r2/g2) so that $1\leq d_{x}$. In the top categorization, $d_{x}=1$, whereas the bottom categorization maximizes $d_{x}$. At any categorical boundary, *x*1 and *x*2 must be different. Otherwise, $d_{x}$ is unsolvable. Figure 2 shows a graph for $d_{x}=(40/30)((70/x)-1)$ in which *x*1s for the bottom, observed, and top categorizations are 10, 25, and 40, respectively. The $d_{x}$ graph can be drawn only in quadrant I; that is, only positive real numbers can be observations.

### HA-coefficient algorithm

Given Equation 1, let us designate the area delimited between *x*1s at the bottom and top categorizations as *W* and the area delimited between *x*1s at the bottom and observed categorizations as *R*. The *W* and *R* represent cumulative hierarchical association distances and can be calculated as follows:

(2) $$W=\frac{g1}{g2}\int_{\mathrm{btm}}^{\mathrm{top}}\left(\frac{y}{x}-1\right)\;dx\;=\frac{g1}{g2}{\left[y\;\ln(x)-x\right]}_{\mathrm{btm}}^{\mathrm{top}}$$

(3) $$R=\frac{g1}{g2}\int_{\mathrm{btm}}^{\mathrm{obs}.}\left(\frac{y}{x}-1\right)dx\;=\frac{g1}{g2}{\left[y\ln(x)-x\right]}_{\mathrm{btm}}^{\mathrm{obs}.}$$

Ultimately, the HA-coefficient can be calculated as follows:

(4) $$\mathrm{HA}=\frac{R}{W}=\frac{{\left[y\;\ln(x)-x\right]}_{\mathrm{btm}}^{\mathrm{obs}.}}{{\left[y\;\ln(x)-x\right]}_{\mathrm{btm}}^{\mathrm{top}}}$$

where HA is the HA-coefficient, *W* is the area delimited between *x*1s in the bottom and top categorizations, *R* is the area delimited between *x*1s in the bottom and observed categorizations, *g*1 is the *x*1 in the top categorization, *g*2 is the *x*2 in the top categorization, *y* is the sum of all observations, *x* is the variable, obs. is the *x*1 in the observed categorization, btm is the *x*1 in the bottom categorization, and top is the *x*1 in the top categorization.

It is always true that 0 ≤ *R* ≤ *W* so that the HA-coefficient results in a proportion. If *x*1 in the observed categorization equals *x*1 in the bottom categorization, HA-coefficient = 0. If *x*1 in the observed categorization equals *x*1 in the top categorization, HA-coefficient = 1. Equation 4 calculates an HA-coefficient if the whole population set consists of 2 categories. If the whole population set consists of equal to or more than 2 categories, either of the following 2 algorithms can be used:

1. HA-coefficient algorithm based on geometric mean

(5) $$\mathrm{HA}=\sqrt[{n-1}]{\overset{n-1}{\underset{k=1}{\prod }}\frac{{[y\;\ln(x)-x]}_{{\mathrm{btm}}_{\left[k\right]}}^{\mathrm{obs}._{\left[k\right]}}}{{[y\;\ln(x)-x]}_{{\mathrm{btm}}_{\left[k\right]}}^{{\mathrm{top}}_{\left[k\right]}}}}$$

1. HA-coefficient algorithm based on arithmetic mean

(6) $$\mathrm{HA}=\frac{\sum_{k=1}^{n-1}\frac{{\left[y\ln(x)-x\right]}_{{\mathrm{btm}}_{\left[k\right]}}^{\mathrm{obs}._{\left[k\right]}}}{{\left[y\ln(x)-x\right]}_{{\mathrm{btm}}_{\left[k\right]}}^{{\mathrm{top}}_{\left[k\right]}}}}{n-1}$$

where HA is the HA-coefficient, *n* is the total number of categories, *k* is the loop variable, *y* is the sum of all observations, *x* is the variable, obs.[*k*] is the *x*1 in the observed categorization given the *k*th categorical boundary, btm[*k*] is the *x*1 in the bottom categorization given the *k*th categorical boundary, and top[*k*] is the *x*1 in the top categorization given the *k*th categorical boundary.

Equations 5 and 6 produce closely similar results. I recommend Equation 5 because unification of the equation is foundational in comparing multiple HA-coefficients. If 2 or more categories have the same average, the HA-coefficient algorithm is not applicable.

### Prehierarchical and posthierarchical categorizations

Hierarchical stratification among categories can be determined independent of or dependent on observations.

#### Definition 6

If hierarchical stratification among categories is determined independent of observations, categories are “prehierarchical.” If hierarchical stratification among categories is determined dependent on observations, categories are “posthierarchical.”

Prehierarchical categorization makes it feasible that HA-coefficient = 0, whereas posthierarchical categorization does not.

### Simulations

**Figure 3.**
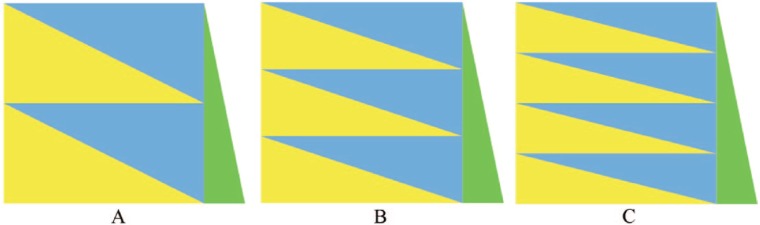
Three simulated data sets of 1200 by 1201. Green triangles refer to a vector containing 1200 observations increasing by 1 from 1001 to 2200. The 1200 by 1200 squares in (A), (B), and (C) are filled with categorical identifiers of 2, 3, and 4 types, respectively. In each matrix, the first blue triangle is filled with 0s, the next blue triangle is filled with 1s, and so on. The yellow triangles are filled with random categorical identifiers.

To demonstrate robustness and reliability of the HA-coefficient algorithm, simple simulations were used. Figures 3A, 3B, and 3C refer to matrices of 1200 by 1201. The green triangle refers to the 1201st column, including 1200 natural numbers increasing by 1 from 1001 to 2200. Figures 3A, 3B, and 3C include 2, 3, and 4 couples of blue and yellow triangles, respectively. In each matrix, blue and yellow triangles are equal in shape and area. The number of blue triangles in each matrix equals the number of types of categorical identifiers. Figures 3A, 3B, and 3C have categorical identifiers of 2 (0, 1), 3 (0, 1, 2), and 4 (0, 1, 2, 3) types, respectively. In each matrix, the top blue triangle is filled with 0s, the next blue triangle is filled with 1s, and so on. The yellow triangles are filled with random categorical identifiers. As a column coordinate *n* changes from 1 to 1200, the HA-coefficient between *n*th and 1201st columns gradually increases to 1. The minimum HA-coefficient must be greater than 0 because each categorization is posthierarchical. The 100 times simulations were averaged into smooth plots and aim to answer the following questions:

*Question 1*. Do the HA-coefficients from Figures 3A, 3B, and 3C increase from left to right?
*Question 2*. Do the HA-coefficients from Figures 3A, 3B, and 3C coincide?
*Question 3.* Do the HA-coefficients and *P* values calculated by the *F* test show a consistent pattern?

Regarding Question 3, the linear model (LM) for the *F* test was set as:

(7) $$y_{ij}=\mu +\alpha_{i}+\varepsilon_{ij}.$$

*i* = 1,2,3, …, *a*

*j* = 1,2,3, …, *n*

where *y*_ij_ is the *j*th observation for *i*th category, *µ* is the mean of all observations, $\alpha_{i}$ is the constant for *i*th category based on random deviation from *µ*, and $\varepsilon_{ij}$is the random effect containing all uncontrolled sources of variability.^5^

Through 100 times simulations, the resulting *P values* were averaged into smooth plots. If the answers to all questions are positive, the HA-coefficient algorithm is reliable and robust. All computations were conducted using R.^6^ All R scripts are included in Supplementary R scripts.

## Results and Discussion

**Figure 4.**
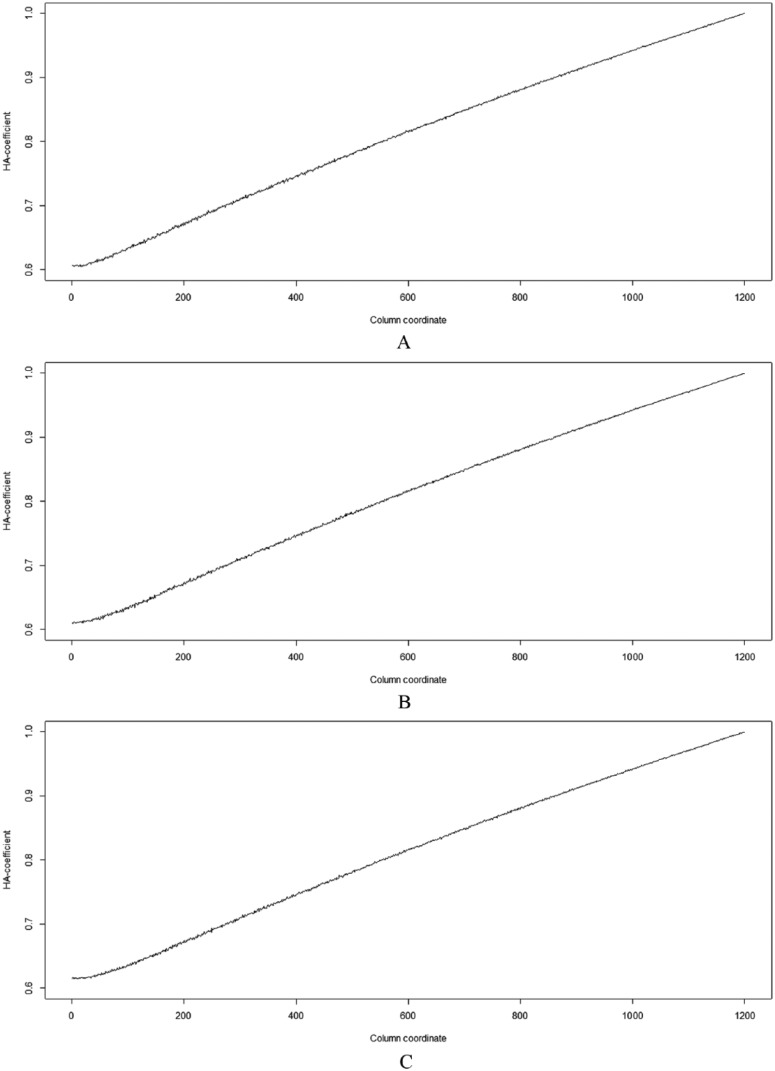
Plots (A), (B), and (C) represent patterns of the HA-coefficients obtained by applying the HA-coefficient algorithm to data sets in Figures 3A, 3B, and 3C, respectively.

**Figure 5.**
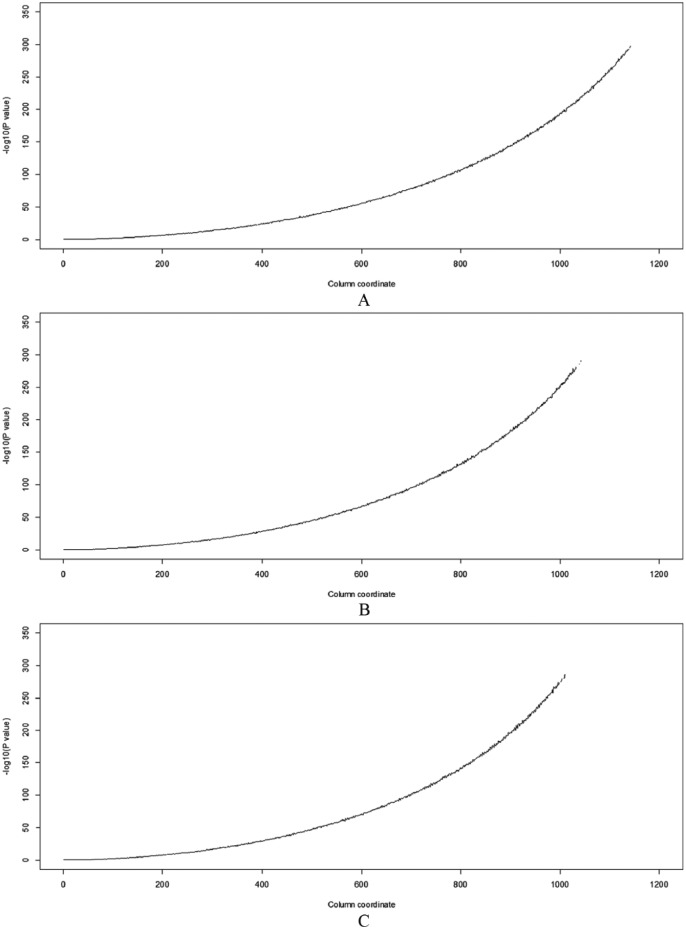
Plots (A), (B), and (C) represent patterns of the *P* values obtained by applying *F* test to data sets in Figures 3A, 3B, and 3C, respectively.

Figure 4 shows 3 plots obtained by applying the HA-coefficient algorithm to data sets in Figures 3A, 3B, and 3C. This illustrates a common increasing pattern and gives a positive answer to Question 1. Each plot ranges between about 0.6 and 1.0. Because the simulated data sets are posthierarchical, it is infeasible that HA-coefficient = 0. The 3 different simulated data sets have the same observations at regular intervals and equal proportions of blue and yellow sections when comparing the same columns. Therefore, the 3 simulated data sets have the same pattern in terms of the association between categories and observations. If the HA-coefficient algorithm is robust and reliable, the same result must be produced from the 3 simulated data sets. Table 1 shows the Pearson correlation coefficients among the 3 plots coincide. This gives a positive answer to Question 2. The increasing pattern of all plots in Figures 4 and 5 gives a positive answer to Question 3. All answers to the above questions are positive. This indicates that the HA-coefficient algorithm is robust and reliable. The curves generated by the *F* test in Figure 5 are bent downward because the −log_10_ lifts small *P* values upward but pushes moderate *P* values downward. The *F* test (see Equation 7) has the following constraints:

*Constraint 1*. Given the top categorization, *P values* = 0. It is impossible to represent −log_10_ (0).
*Constraint 2*. Three assumptions for the LM are required: (1) $\varepsilon_{ij}s$ conform the normal distribution, (2) $\varepsilon_{ij}s$ have the same variance for each *i*, and $\varepsilon_{ij}s$ are independent of each other and the $\alpha_{i}s.$^5^

**Table 1.** Pearson correlation coefficients among the 3 plots in Figure 4.

	Figure 3A	Figure 3B	Figure 3C
Figure 3A	1	0.999893	0.999845
Figure 3B	0.999893	1	0.999883
Figure 3C	0.999845	0.999883	1

The above constraints do not apply to the HA-coefficient algorithm. Regarding Constraint 1, the graph lines obtained by the *F* test (Figure 5) do not reach the right end, while graph lines obtained by the HA-coefficient algorithm (Figure 4) are fully drawn from left to right ends. Regarding Constrain 2, the HA-coefficient algorithm produces an objective measurement; that is, assumptions for statistical inference are not needed. The simulations revealed that the HA-coefficient algorithm is faster than the *F* test based on the LM, e.g. when applied to Figure 3A, the former and the latter took 739 and 956 seconds (Intel i7-5600U CPU), respectively.

## Conclusion

This study shows a comparison of the HA-coefficient algorithm and *F* test because both methods calculate the association between categories and observations based on the degree of variance among averages for all categories. The HA-coefficient algorithm’s objectivity, reliability, robustness, and speed enable the algorithm to become an alternative to the *F* test. When it comes to GWAS, the HA-coefficient algorithm will be suited for a population grown in the same environment because the same environment is fundamental in identifying unbiased QTL. Posthierarhichical categorizations are shown by the data sets in Figure 3. GWAS data sets have the posthierarchical categorization. The application of the HA-coefficient algorithm to a prehierarchical categorization is shown in Supplementary example. The HA-coefficient algorithm will be useful in many disciplines.

## Supplementary Material

Supplementary material

Supplementary material
