# Supplementary material for: Hierarchical Association Coefficient Algorithm: New Method for Genome-Wide Association Study
Source: Evol Bioinform Online. 2017 Aug 31;13:1176934317713004. doi: 10.1177/1176934317713004 (PMC5582720; doi:10.1177/1176934317713004)
Supplement: Supplementary material [file Supplementary_example.pdf]

Supplementary example

Application of the HA-coefficient algorithm to prehierarchical categorization

Table 1 shows scores from two Mathematics quizzes for the same 27 students. Based on scores from Quiz 1, a teacher classifies all students into three categories:

Category 3: 70 ≤ score

Category 2: 60 ≤ score < 70

Category 1: score < 60

New scores from a subsequent quiz for the same students are often differently ranked. At that time, the teacher could raise the following question: how much of categorical stratification based on Quiz 1 is maintained in the results of Quiz 2? In order to answer this question, the categorization determined based on Quiz 1 must be fixed and applied to the scores of Quiz 2. The scores of Quiz 2 can be resorted into the top and bottom categorizations, which are shown in Table 2. Table 3 shows *x1*s and *x2*s at each categorical boundary. Given the example, a HA-coefficient can be calculated by substituting the sum of the total observations and *x1*s from Table 3 for *y* and *x*, respectively, in Equation 5:

$$\mathrm{HA} = \sqrt{\frac{[\,1771\ln(x) - x]_{1003}^{1136}}{[\,1771\ln(x) - x]_{1003}^{1234}} \cdot \frac{[\,1771\ln(x) - x]_{415}^{572}}{[1771\ln(x) - x]_{415}^{626}}} = 0.715$$

where HA = the HA-coefficient; *x* = the variable.

According to the above result, the new categorization from Quiz 2 maintains 71.5 % of categorization determined by Quiz 1.

Table 1. Scores of the two Mathematics quizzes for 27 students. The three categories are classified based on the scores from Quiz 1, in which Categories 3, 2, and 1 satisfy conditions: 70 ≤ score, 60 ≤ score < 70, and score < 60, respectively.

**Category**	**ID**	**Mathematics score**	
		**Quiz 1**	**Quiz 2**
Category 3 (C3)	Student.1	88	79
	Student.2	83	82
	Student.3	81	80
	Student.4	87	73
	Student.5	78	70
	Student.6	71	52
	Student.7	71	75
	Student.8	75	61
Category 2 (C2)	Student.9	69	57
	Student.10	68	77
	Student.11	68	67
	Student.12	67	59
	Student.13	66	54
	Student.14	66	46
	Student.15	64	66
	Student.16	62	53
	Student.17	61	85
Category 1 (C1)	Student.18	58	68
	Student.19	58	69
	Student.20	57	51
	Student.21	56	61
	Student.22	55	72
	Student.23	52	61
	Student.24	52	67
	Student.25	48	75
	Student.26	50	68
	Student.27	49	43

Table 2. Resorted scores of the Mathematics Quiz 2 according to the observed, top, and bottom categorizations.

**Category**	**ID**	**Mathematics score**			
		**Quiz 1**	**Quiz 2**		
			**Observed**	**Top**	**Bottom**
Category 3 (C3)	Student.1	88	79	85	43
	Student.2	83	82	82	46
	Student.3	81	80	80	51
	Student.4	87	73	79	52
	Student.5	78	70	77	53
	Student.6	71	52	75	54
	Student.7	71	75	75	57
	Student.8	75	61	73	59
Category 2 (C2)	Student.9	69	57	72	61
	Student.10	68	77	70	61
	Student.11	68	67	69	61
	Student.12	67	59	68	66
	Student.13	66	54	68	67
	Student.14	66	46	67	67
	Student.15	64	66	67	68
	Student.16	62	53	66	68
	Student.17	61	85	61	69
Category 1 (C1)	Student.18	58	68	61	70
	Student.19	58	69	61	72
	Student.20	57	51	59	73
	Student.21	56	61	57	75
	Student.22	55	72	54	75
	Student.23	52	61	53	77
	Student.24	52	67	52	79
	Student.25	48	75	51	80
	Student.26	50	68	46	82
	Student.27	49	43	43	85

Table 3. The *x1*s and *x2*s calculated from Table 2. The first column shows the three categories (C1, C2, and C3) in ascending order based on the mean of the observations in each category, in which a vertical bar refers to a boundary used to group the whole population set into two subsets (*x2* and *x1*). The parentheses collapse multiple categories into the same subset.

**Grouping of *x1* and *x2***	**Categorization**	***x1***	***x2***	**Total sum**
C1 | (C2, C3) [*x2* | *x1*]	Observed	1136	635	1771
	Top	1234	537	1771
	Bottom	1003	768	1771
(C1, C2) | C3 [*x2* | *x1*]	Observed	572	1199	1771
	Top	626	1145	1771
	Bottom	415	1356	1771
