# Supplementary material for: Hierarchical Association Coefficient Algorithm: New Method for Genome-Wide Association Study
Source: Evol Bioinform Online. 2017 Aug 31;13:1176934317713004. doi: 10.1177/1176934317713004 (PMC5582720; doi:10.1177/1176934317713004)
Supplement: Supplementary material [file Supplementary_R_scripts.pdf]

Supplementary R scripts

```
###################
#   Figure 3a
###################

sum_vec <- 0
ptm1 <- proc.time()
for (k in 1:100){
  dat <- matrix( c(rep(3,1440000)),nrow=1200,ncol=1200)
  vec <- seq(1001,2200,by=1)
  for (i in 1:1200){
    for (j in 1:1200){
      dat[i,j] <- sample(0:1,1)
  }}
  for (i in 1:1200){
    for (j in 1:floor(0.5*i)){
      dat[j,i] <- 0
    }
    for (j in 601:(600 + floor(0.5*i)) ){
      dat[j,i] <- 1
    }
  }
  dat <- cbind(dat,vec)
  res <- 0
  for (i in 1:(length(dat[1,])-1)){
   su  <- 0
   geno  <- dat[,i]
   pheno <- dat[,length(dat[1,])]
   ave_0 <- mean( pheno[geno == 0])
   ave_1 <- mean( pheno[geno == 1])
   ord <- c(ave_0, ave_1)
   ord <- rank(ord)
   ref <- c(0,1)
   who_len  <- length(geno)
   min_len  <- length( which( geno == ref[ which(ord == 1) ]) )
   max_len  <- length( which( geno == ref[ which(ord == 2) ]) )
   i_list <- sort(pheno,decreasing=F)
   top_g0 <- i_list[1 : min_len]
   top_g1 <- i_list[(min_len + 1) : (min_len + max_len) ]
   r_list <- sort( pheno,decreasing=T)
   bot_g0 <- r_list[1 : min_len]
   bot_g1 <- r_list[(min_len + 1) : (min_len + max_len) ]
   top_s0 <- sum(top_g0)
   top_s1 <- sum(top_g1)
   bot_s0 <- sum(bot_g0)
   bot_s1 <- sum(bot_g1)
   obs_s0 <- sum( pheno[which( geno == ref[ which(ord == 1) ])] )
   obs_s1 <- sum( pheno[which( geno == ref[ which(ord == 2) ])] )
```

```
   su <- obs_s0 + obs_s1

   x2 <- (su*log(obs_s1) - obs_s1) - (su*log(bot_s1) - bot_s1)
   x1 <- (su*log(top_s1) - top_s1) - (su*log(bot_s1) - bot_s1)
   res_1 <- x2/x1

   res[i] <- res_1
}
sum_vec <- sum_vec + res
}
ptm2 <- proc.time()
cat(ptm2-ptm1)
plot(sum_vec/100,type="l",ylab = "HA-coefficient",xlab = "Column
coordinate",ylim=c(0.6,1.0))

###################
#   Figure 3b
###################

sum_vec <- 0
ptm1 <- proc.time()
for (k in 1:100){
  dat <- matrix( c(rep(3,1440000)),nrow=1200,ncol=1200)
  vec <- seq(1001,2200,by=1)
  for (i in 1:1200){
    for (j in 1:1200){
      dat[i,j] <- sample(0:2,1)
  }}
  for (i in 1:1200){
    for (j in 1:floor(0.3334*i)){
      dat[j,i] <- 0
    }
    for (j in 401:(400 + floor(0.3334*i)) ){
      dat[j,i] <- 1
    }
    for (j in 801:(800 + floor(0.3334*i)) ){
      dat[j,i] <- 2
    }
  }
  dat <- cbind(dat,vec)
  res <- 0
  for (i in 1:(length(dat[1,])-1)){
   su  <- 0
   geno  <- dat[,i]
   pheno <- dat[,length(dat[1,])]
   ave_0 <- mean( pheno[geno == 0])
   ave_1 <- mean( pheno[geno == 1])
   ave_2 <- mean( pheno[geno == 2])
```

```
   ord <- c(ave_0, ave_1, ave_2)
   ord <- rank(ord)
   ref <- c(0,1,2)
   who_len <- length(geno)
   min_len <- length( which( geno == ref[ which(ord == 1) ]) )
   mid_len <- length( which( geno == ref[ which(ord == 2) ]) )
   max_len <- length( which( geno == ref[ which(ord == 3) ]) )
   i_list <- sort(pheno,decreasing=F)
   top_g0 <- i_list[ 1 : min_len ]
   top_g1 <- i_list[ (min_len + 1) : (min_len+mid_len) ]
   top_g2 <- i_list[ (min_len+mid_len + 1) : (min_len + mid_len + max_len) ]
   r_list <- sort( pheno,decreasing=T)
   bot_g0 <- r_list[ 1 : min_len ]
   bot_g1 <- r_list[ (min_len + 1) : (min_len+mid_len) ]
   bot_g2 <- r_list[ (min_len+mid_len + 1) : (min_len + mid_len + max_len) ]
   top_s0 <- sum(top_g0)
   top_s1 <- sum(top_g1)
   top_s2 <- sum(top_g2)
   bot_s0 <- sum(bot_g0)
   bot_s1 <- sum(bot_g1)
   bot_s2 <- sum(bot_g2)
   obs_s0 <- sum( pheno[which( geno == ref[ which(ord == 1) ])] )
   obs_s1 <- sum( pheno[which( geno == ref[ which(ord == 2) ])] )
   obs_s2 <- sum( pheno[which( geno == ref[ which(ord == 3) ])] )
   su <- sum(obs_s0,obs_s1,obs_s2)

   x2 <- (su*log(obs_s1 + obs_s2) - (obs_s1 + obs_s2)) - (su*log(bot_s1+bot_s2) -
(bot_s1+bot_s2))
   x1 <- (su*log(top_s1 + top_s2) - (top_s1 + top_s2)) - (su*log(bot_s1+bot_s2) -
(bot_s1+bot_s2))
   res_1 <- x2/x1

   x2 <- (su*log(obs_s2) - obs_s2) - (su*log(bot_s2) - bot_s2)
   x1 <- (su*log(top_s2) - top_s2) - (su*log(bot_s2) - bot_s2)
   res_2 <- x2/x1

   res[i] <-(res_1*res_2)^0.5
}
sum_vec <- sum_vec + res
}
ptm2 <- proc.time()
cat(ptm2-ptm1)
plot(sum_vec/100,type="l",ylab = "HA-coefficient",xlab = "Column
coordinate",ylim=c(0.6,1.0))

###################
#   Figure 3c
###################
```

```
sum_vec <- 0
ptm1 <- proc.time()
for (k in 1:100){
  dat <- matrix( c(rep(3,1440000)),nrow=1200,ncol=1200)
  vec <- seq(1001,2200,by=1)
  for (i in 1:1200){
    for (j in 1:1200){
      dat[i,j] <- sample(0:3,1)
  }}
  for (i in 1:1200){
    for (j in 1:floor(0.25*i)){
      dat[j,i] <- 0
    }
    for (j in 301:(300 + floor(0.25*i)) ){
      dat[j,i] <- 1
    }
    for (j in 601:(600 + floor(0.25*i)) ){
      dat[j,i] <- 2
    }
    for (j in 901:(900 + floor(0.25*i)) ){
      dat[j,i] <- 3
    }
  }
  dat <- cbind(dat,vec)
  res <- 0
  for (i in 1:(length(dat[1,])-1)){
   su  <- 0
   geno  <- dat[,i]
   pheno <- dat[,length(dat[1,])]
   ave_0 <- mean( pheno[geno == 0])
   ave_1 <- mean( pheno[geno == 1])
   ave_2 <- mean( pheno[geno == 2])
   ave_3 <- mean( pheno[geno == 3])
   ord <- c(ave_0, ave_1, ave_2,ave_3)
   ord <- rank(ord)
   ref <- c(0,1,2,3)
   who_len  <- length(geno)
   min_len  <- length( which( geno == ref[ which(ord == 1) ]) )
   mid_len1 <- length( which( geno == ref[ which(ord == 2) ]) )
   mid_len2 <- length( which( geno == ref[ which(ord == 3) ]) )
   max_len  <- length( which( geno == ref[ which(ord == 4) ]) )
   i_list <- sort(pheno,decreasing=F)
   top_g0 <- i_list[1 : min_len]
   top_g1 <- i_list[(min_len + 1) : (min_len+mid_len1)]
   top_g2 <- i_list[(min_len+mid_len1 + 1) : (min_len+mid_len1+mid_len2)]
   top_g3 <- i_list[(min_len+mid_len1+mid_len2 + 1) : (min_len+mid_len1+mid_len2 +
max_len) ]
```

```
   r_list <- sort( pheno,decreasing=T)
   bot_g0 <- r_list[1 : min_len]
   bot_g1 <- r_list[(min_len + 1) : (min_len+mid_len1)]  # Áß°£ Æò±Õ
   bot_g2 <- r_list[(min_len+mid_len1 + 1) : (min_len+mid_len1+mid_len2)]
   bot_g3 <- r_list[(min_len+mid_len1+mid_len2 + 1) : (min_len+mid_len1+mid_len2 +
max_len) ]
   top_s0 <- sum(top_g0)
   top_s1 <- sum(top_g1)
   top_s2 <- sum(top_g2)
   top_s3 <- sum(top_g3)
   bot_s0 <- sum(bot_g0)
   bot_s1 <- sum(bot_g1)
   bot_s2 <- sum(bot_g2)
   bot_s3 <- sum(bot_g3)
   obs_s0 <- sum( pheno[which( geno == ref[ which(ord == 1) ])] )
   obs_s1 <- sum( pheno[which( geno == ref[ which(ord == 2) ])] )
   obs_s2 <- sum( pheno[which( geno == ref[ which(ord == 3) ])] )
   obs_s3 <- sum( pheno[which( geno == ref[ which(ord == 4) ])] )
   su <- obs_s0 + obs_s1 + obs_s2 + obs_s3

   x2 <- (su*log(obs_s1 + obs_s2 + obs_s3) - (obs_s1 + obs_s2 + obs_s3)) -
(su*log(bot_s1+bot_s2+bot_s3) - (bot_s1+bot_s2+bot_s3))
   x1 <- (su*log(top_s1 + top_s2 + top_s3) - (top_s1 + top_s2 + top_s3)) -
(su*log(bot_s1+bot_s2+bot_s3) - (bot_s1+bot_s2+bot_s3))
   res_1 <- x2/x1

   x2 <- (su*log(obs_s2+obs_s3) - (obs_s2+obs_s3)) - (su*log(bot_s2+bot_s3) -
(bot_s2+bot_s3))
   x1 <- (su*log(top_s2+top_s3) - (top_s2+top_s3)) - (su*log(bot_s2+bot_s3) -
(bot_s2+bot_s3))
   res_2 <- x2/x1

   x2 <- (su*log(obs_s3) - obs_s3) - (su*log(bot_s3) - bot_s3)
   x1 <- (su*log(top_s3) - top_s3) - (su*log(bot_s3) - bot_s3)
   res_3 <- x2/x1

   res[i] <-(res_1*res_2*res_3)^(1/3)
}
sum_vec <- sum_vec + res
}
ptm2 <- proc.time()
cat(ptm2-ptm1)
plot(sum_vec/100,type="l",ylab = "HA-coefficient",xlab = "Column
coordinate",ylim=c(0.6,1.0))

###################
#   Figure 4a
###################
```

```
sum_vec <- 0
ptm1    <- proc.time()

for (k in 1:100){
  d <- matrix( c(rep(0,1440000)),nrow=1200,ncol=1200)
  vec <- seq(1001,2200,by=1)
  for (i in 1:1200){
    for (j in 1:1200){
      d[i,j] <- sample(0:1,1,replace=T)
  }}
  for (i in 1:1200){
    for (j in 1:floor(0.5*i)){
      d[j,i] <- 0
    }
    for (j in 601:(600 + floor(0.5*i)) ){
      d[j,i] <- 1
    }
  }
  d <- cbind(d,vec)
  d <- data.frame(d)
   res <- 0
   vec <- 0
   for (i in 1:1200){
     res <- summary(lm(d[,1201] ~ 1 + d[,i],data = d))
     vec[i] <- res$coefficients[2,4]
   }
   pv <- -1 * log10(vec)

sum_vec <- sum_vec + pv
}
ptm2 <- proc.time()
cat(ptm2-ptm1)
plot(sum_vec/100,type="l",ylab = "log10(p-value)",xlab = "Column
coordinate",ylim=c(0,350))

###################
#   Figure 4b
###################

sum_vec <- 0
ptm1    <- proc.time()

for (k in 1:100){
  d <- matrix( c(rep(0,1440000)),nrow=1200,ncol=1200)
  vec <- seq(1001,2200,by=1)
  for (i in 1:1200){
    for (j in 1:1200){
```

```
      d[i,j] <- sample(0:2,1,replace=T)
  }}
  for (i in 1:1200){
    for (j in 1:floor(0.3334*i)){
      d[j,i] <- 0
    }
    for (j in 401:(400 + floor(0.3334*i)) ){
      d[j,i] <- 1
    }
    for (j in 801:(800 + floor(0.3334*i)) ){
      d[j,i] <- 2
    }
  }
  d <- cbind(d,vec)
  d <- data.frame(d)
   res <- 0
   vec <- 0
   for (i in 1:1200){
     res <- summary(lm(d[,1201] ~ 1 + d[,i],data = d))
     vec[i] <- res$coefficients[2,4]
   }
   pv <- -1 * log10(vec)

sum_vec <- sum_vec + pv
}
ptm2 <- proc.time()
cat(ptm2-ptm1)
plot(sum_vec/100,type="l",ylab = "log10(p-value)",xlab = "Column
coordinate",ylim=c(0,350))

###################
#   Figure 4c
###################

sum_vec <- 0
ptm1    <- proc.time()

for (k in 1:100){
  d <- matrix( c(rep(0,1440000)),nrow=1200,ncol=1200)
  vec <- seq(1001,2200,by=1)
  for (i in 1:1200){
    for (j in 1:1200){
      d[i,j] <- sample(0:3,1,replace=T)
  }}
  for (i in 1:1200){
    for (j in 1:floor(0.25*i)){
      d[j,i] <- 0
    }
```

```
    for (j in 301:(300 + floor(0.25*i)) ){
      d[j,i] <- 1
    }
    for (j in 601:(600 + floor(0.25*i)) ){
      d[j,i] <- 2
    }
    for (j in 901:(900 + floor(0.25*i)) ){
      d[j,i] <- 3
    }
  }
  d <- cbind(d,vec)
  d <- data.frame(d)
   res <- 0
   vec <- 0
   for (i in 1:1200){
     res <- summary(lm(d[,1201] ~ 1 + d[,i],data = d))
     vec[i] <- res$coefficients[2,4]
   }
   pv <- -1 * log10(vec)

sum_vec <- sum_vec + pv
}
ptm2 <- proc.time()
cat(ptm2-ptm1)
plot(sum_vec/100,type="l",ylab = "log10(p-value)",xlab = "Column
coordinate",ylim=c(0,350))


##############################
#   Supplementary example
##############################

   quiz_1 <-
c(88,83,81,87,78,71,71,75,69,68,68,67,66,66,64,62,61,58,58,57,56,55,52,52,48,50,49)
   quiz_2 <-
c(79,82,80,73,70,52,75,61,57,77,67,59,54,46,66,53,85,68,69,51,61,72,61,67,75,68,43)
   d    <- cbind(quiz_1,quiz_2)

   i_list <- sort(d[,2],decreasing=T)
   top_g2 <- i_list[1:8]
   top_g1 <- i_list[9:17]
   top_g0 <- i_list[18:27]
   r_list <- rev( i_list )
   bot_g2 <- r_list[1:8]
   bot_g1 <- r_list[9:17]
   bot_g0 <- r_list[18:27]
   top_s2 <- sum(top_g2)
   top_s1 <- sum(top_g1)
```

```
   top_s0 <- sum(top_g0)
   bot_s2 <- sum(bot_g2)
   bot_s1 <- sum(bot_g1)
   bot_s0 <- sum(bot_g0)
   obs_s2 <- sum(d[,2][1:8])
   obs_s1 <- sum(d[,2][9:17])
   obs_s0 <- sum(d[,2][18:27])
   su <- sum(obs_s0,obs_s1,obs_s2)

   x2 <- (su*log(obs_s1 + obs_s2) - (obs_s1 + obs_s2)) - (su*log(bot_s1+bot_s2) -
(bot_s1+bot_s2))
   x1 <- (su*log(top_s1 + top_s2) - (top_s1 + top_s2)) - (su*log(bot_s1+bot_s2) -
(bot_s1+bot_s2))
   res_1 <- x2/x1

   x2 <- (su*log(obs_s2) - obs_s2) - (su*log(bot_s2) - bot_s2)
   x1 <- (su*log(top_s2) - top_s2) - (su*log(bot_s2) - bot_s2)
   res_2 <- x2/x1

   res <-(res_1*res_2)^0.5
res
```
